# Compound heterozygous missense and intronic variants in *B9D1* contribute to a recurrent Meckel syndrome pedigree

**DOI:** 10.3389/fgene.2025.1663455

**Published:** 2025-08-26

**Authors:** Huining Jing, Bocheng Xu, Hao Wang, Shanling Liu, He Wang, Jingqun Mai, Wencong Yao, Zhu Zhang

**Affiliations:** ^1^ Department of Medical Genetics, West China Second University Hospital, Sichuan University, Chengdu, China; ^2^ Key Laboratory of Birth Defects and Related Diseases of Women and Children (Sichuan University), Ministry of Education, Chengdu, China

**Keywords:** Meckel syndrome, *B9D1*, whole exome sequencing, minigene splicing assay, reanalysis

## Abstract

**Background:**

Meckel syndrome (MKS) is an embryonically lethal ciliopathy with severe clinical manifestations, including defects of the central nervous system, bilateral renal cystic dysplasia, and postaxial polydactyly. B9 domain-containing 1 (B9D1, NP_056496.1) is a member of a small family of proteins associated with basal bodies and primary cilia in mammalian cells. *B9D1* variants are associated with MKS and Joubert syndrome. However, to date, only a few cases have been reported.

**Methods:**

In this study, we investigated a prenatally diagnosed recurrent MKS pedigree. Two fetuses of different sexes were conceived by nonconsanguineous parents. Systematic color Doppler ultrasound revealed same malformations in both fetuses during the second trimester, which included meningoencephalocele, Dandy-Walker malformation, and postaxial polydactyly. Trio whole exome sequencing (WES) and WES reanalysis were performed. The presence and effects of these variants were further validated using Sanger sequencing, RT-PCR, and minigene splicing assay at the DNA and RNA levels.

**Results:**

Two compound heterozygous variants, c.341G>T (p.R114L) and c.405-308_405-304del, were identified in both probands, each inherited from one unaffected parent. Both variants led to abnormal splicing. Specifically, the missense mutation c.341G>T caused the skipping of exon 4, whereas the novel deep-intronic variant c.405-308_405-304del created a new and strong acceptor site at c.405-294_405-293. Pathogenicity analysis indicated that both variants were pathogenic.

**Conclusion:**

This report presents a rare pedigree of recurrent MKS, in which two novel mutations in *B9D1* are identified. Our findings expand the mutation spectrum of *B9D1* and provide an accurate molecular diagnosis for genetic counseling.

## Introduction

B9 domain-containing 1 (*B9D1*, NP_056496.1) belongs to a small family of proteins associated with basal bodies and primary cilia in mammalian cells (Bialas et al., 2009). Proteins within this family are hypothesized to play pivotal roles in cilia formation and retention (Dowdle et al., 2011). All the three family members, such as *B9D1* (OMIM: 614144), B9D2 (OMIM: 611951), and MKS transition zone complex subunit 1 (MKS1, OMIM: 609883), possess the B9 domain and are localized to ciliary axonemes and basal bodies in ciliated mouse IMCD3 cells and to centrosomes in non-ciliated mouse IMCD3 cells (Chih et al., 2011).

*B9D1* (NM_015681.6) is located on chromosome 17p11.2 and has an open reading frame of 1,059 base pairs. As of September 2024, 16 mutations in *B9D1* have been reported in the Human Gene Mutation Database (HGMD) (Stenson et al., 2003). In ciliopathy, functional and structural defects of the cilium have been identified as the causative factors of inherited diseases with multiorgan phenotypes. Most proteins altered in these single-gene disorders function at the level of the cilium-centrosome complex (Hildebrandt et al., 2011).

MKS is a rare (1:10,000–1:140,000) and embryonically lethal autosomal recessive disease (Barisic et al., 2015). It was first described by Johann Friedrich Meckel in 1822. MKS is the most severe ciliopathy. Its clinical features include defects in the central nervous system (considered obligatory features of MKS, but presenting variably, typically occipital encephalocele, Dandy-Walker malformation, and hydrocephalus), postaxial polydactyly (observed in 70%–80% of cases), bilateral renal cystic dysplasia, polycystic kidney disease, and congenital hepatic fibrosis (Logan et al., 2011; Summers and Donnenfeld, 1995; Hartill et al., 2017; Turkyilmaz et al., 2021). MKS has genetic and phenotypic overlap with JS, a viable ciliopathy (Parisi, 2019; Tallila et al., 2009).

Here, we report a recurrent pedigree with biallelic pathogenic variants of *B9D1* and a clinical and radiological phenotype consistent with Meckel syndrome.

## Materials and methods

### Clinical data

The mother of the probands, a 37-year-old female, had sequential pregnancies with two malformed fetuses. Family disease history was investigated, and imaging analyses of the two pregnancies were performed. All family members involved in this study and the investigator signed written informed consent forms regarding genetic testing, research, and publication of the relevant data. This study was approved by the Medical Ethics Committee of West China Second University Hospital. Clinical and laboratory examinations were conducted on the probands after written informed consent was obtained from their parents. All procedures were performed in accordance with the Declaration of Helsinki.

### Trio whole exome sequencing (WES) to screen the variants

For DNA extraction, muscle tissue samples from the second proband (aborted fetus) and blood samples from their parents were obtained and processed using a QIAamp DNA Blood Mini Kit (Qiagen, Shanghai, China), following the manufacturer’s instructions. The DNA from the first fetus was utilized directly, as amniocentesis was performed during the mother’s initial pregnancy, allowing for the extraction and preservation of DNA at −80 °C. Exome capture sequencing was performed using NanoWES Human Exome V1 (Berry Genomics, Beijing, China), according to the manufacturer’s instructions. The enriched library was sequenced on an Illumina NovaSeq6000 platform (Illumina Inc., San Diego, CA, United States), generating 150-bp paired-end reads. Burrows–Wheeler Aligner was used to align the sequencing reads with hg38. Local alignment and base quality recalibration of the Burrows–Wheeler aligned reads were performed using GATK Indel Realigner and GATK Base Recalibrator, respectively (broadinstitute.org/). Single-nucleotide variants (SNVs) and small insertions or deletions (InDels) were identified using GATK Unified Genotyper (broadinstitute.org/). Functional annotation was performed using ANNOVAR and Enliven Variants Annotation Interpretation System (Berry Genomics). Public databases for filtering included gnomAD (http://gnomad.broadinstitute.org/) and the 1000 Genomes Project (1000G) (http://browser.1000genomes.org). The pathogenicity of SNVs was evaluated based on scientific medical literature and disease databases such as PubMed (https://www.ncbi.nlm.nih.gov/pubmed/), ClinVar (http://www.ncbi.nlm.nih.gov/clinvar), OMIM (http://www.omim.org), HGMD (http://www.hgmd.org), and the Human Genome Variation Society website (http://www.hgvs.org/dblist/dblist.html). Potential disease-causing variants associated with the patient-standardized Human Phenotype Ontology phenotype were prioritized. The pathogenicity of the novel variants was assessed using SIFT, PolyPhen-2, MutationTaster, SpliceAI, and REVEL. Additionally, the variations reported in HGMD and ClinVar were further examined.

### Sanger sequencing to verify the variants

The likely pathogenic variants identified in the probands by WES were validated by Sanger sequencing using specific primers. The reference sequence NM_015681.6 of *B9D1* was used. Sanger validation primer sets were designed using Primer Premier v.6.0. Amplification by polymerase chain reaction (PCR) was performed using GoldStar Best Master Mix (Cwbio Science, Taizhou, Jiangsu, China). The PCR products were analyzed by 2%–2.5% agarose gel electrophoresis to determine the fragment size. Subsequently, they were purified using a Gel Extraction Kit (Cwbio Science) and sequenced using an ABI 3500×L Dx automated sequencer (Thermo Fisher Scientific, Agawam, MA, United States of America). Sequencing Analysis v.5.2 and Chromas software were used for sequence analysis.

### RNA extraction, reverse transcription PCR (RT–PCR), and Sanger sequencing

Total RNA was extracted from peripheral blood of the parents using TRIzol reagent (Invitrogen, Carlsbad, CA, United States of America), according to the manufacturer’s instructions. RNA was reverse-transcribed using a PrimeScript RT Reagent Kit (Takara Bio Inc., Beijing, China) according to the manufacturer’s instructions. The sequenced fragments of *B9D1* were amplified from cDNA of the parents using forward and reverse primers (Table 1). Subsequently, cDNA alterations were detected by Sanger sequencing.

**TABLE 1:** The primers used in amplification of target fragments and Minigene assay.

Primers	Forward (5′–3′)	Reversed (5′–3′)	Applications
B9D1-341-F/B9D1-341-R	GCACTGGTGTGGAACTTC	GAC​GTA​GAT​TCT​GGG​ACA​AA	Splicing Study for paternal variant B9D1 c.341G>T
17115-B9D1-F/19915-B9D1-R	AAG​GTC​ACG​TGC​ACG​GTT​TC	ACC​TTC​CTC​TCC​CTG​CCA​AGG	Minigene assay for maternal variant B9D1 c.405-308_405-304del
17368-B9D1-F/19655-B9D1-R	GTT​CCC​TGC​AGT​CTG​AGG​GGT	GGT​GCC​ATG​CCC​AGT​TAG​TT	
14984-B9D1-F/15958-B9D1-R	GTG​GGT​TGG​GGA​GGT​AGT​GG	TTC​ATG​CTG​GGG​CCT​GCA​TCC	
pcDNA3.1-B9D1-KpnI-F/pcDNA3.1-B9D1-EcoRI-R	GCT​TGG​TAC​CAT​GCG​GCA​CAA​AAG​GAC​CAT​CCC​CA	TGC​AGA​ATT​CTC​TCT​GAA​AAT​GAG​ACT​TTA	
pcDNA3.1-B9D1-joint-F/pcDNA3.1-B9D1-joint-R	TGC​CCT​TTA​CAT​GTG​GGG​TGC​TGT​GAG​AGT​C	GAC​TCT​CAC​AGC​ACC​CCA​CAT​GTA​AAG​GCA	
B9D1-mut-F/B9D1-mut-R	CCC​CAG​AAA​TCT​TCC​TCT​CAT​TCC​CAG​TGA​A	TTC​ACT​GGG​GAA​TGA​GAG​GAA​GAT​TCT​GGG​G	
pcDNA3.1-F/pcDNA3.1-R	CTA​GAG​AAC​CCA​CTG​CTT​AC	TAGAAGGCACAGTCGAGG	

### Minigene splicing assay

An *in vitro* minigene splicing assay was performed to verify the potential splicing effects of the c.405-308_405-304del variant. The minigene construction strategy for pcDNA3.1-B9D1-wt/mut was the insertion of exon5 (63 bp)-partial intron5 (1,491 bp)-exon6 (68 bp)-intron6 (328 bp)-exon7 (287 bp) into pcDNA3.1. Two pairs of nested primers, 17115-B9D1-F and 19915-B9D1-R, and 17368-B9D1-F and 19655-B9D1-R, were designed. Nested PCR was performed using normal human genomic DNA as a template. A pair of primers, 14984-B9D1-F and 15958-B9D1-R, was designed. PCR was performed using normal human genomic DNA as a template to obtain product one. The left half fragment (472 bp) of *B9D1* wild-type recombinant vector of pcDNA3.1 was amplified using product one as the template and pcDNA3.1-B9D1-KpnI-F and pcDNA3.1-B9D1-joint-R as primers. The right half fragment (1,820 bp) was amplified using the product from the second round of nested PCR as a template and pcDNA3.1-B9D1-EcoRI-R and pcDNA3.1-B9D1-joint-F as primers. Using equally mixed left- and right-half fragments as templates and pcDNA3.1-B9D1-KpnI-F and pcDNA3.1-B9D1-EcoRI-R as primers, *B9D1* wild-type recombinant vectors of pcDNA3.1 (2,262 bp) were amplified. Subsequently, the above steps were repeated to amplify the left half (1,281 bp) and right half (1,016 bp) fragments of *B9D1* mutant recombinant vectors of pcDNA3.1, using pcDNA3.1-B9D1-KpnI-F/B9D1-mut-R, pcDNA3.1-B9D1-EcoRI-R/B9D1-mut-F as primers, and diluted wild-type plasmid as a template. Using equally mixed left and right half fragments as templates, and pcDNA3.1-B9D1-KpnI-F and pcDNA3.1-B9D1-EcoRI-R as primers, *B9D1* mutant recombinant vector of pcDNA3.1 (2,262 bp) was amplified.

The recombinant vectors were transiently transfected into HeLa and 293T cells. After 48 h, cells were collected. Total RNA was extracted from cells, and RT–PCR was performed to synthesize cDNA. Flanking primers (pcDNA3.1-F/pcDNA3.1-B9D1-EcoRI-R) were used for PCR amplification. Amplified gene fragments were detected by agarose gel electrophoresis. Each band was subjected to Sanger sequencing to determine the presence of abnormally spliced isoforms.

## Results

### Clinical phenotype

**FIGURE 1:**
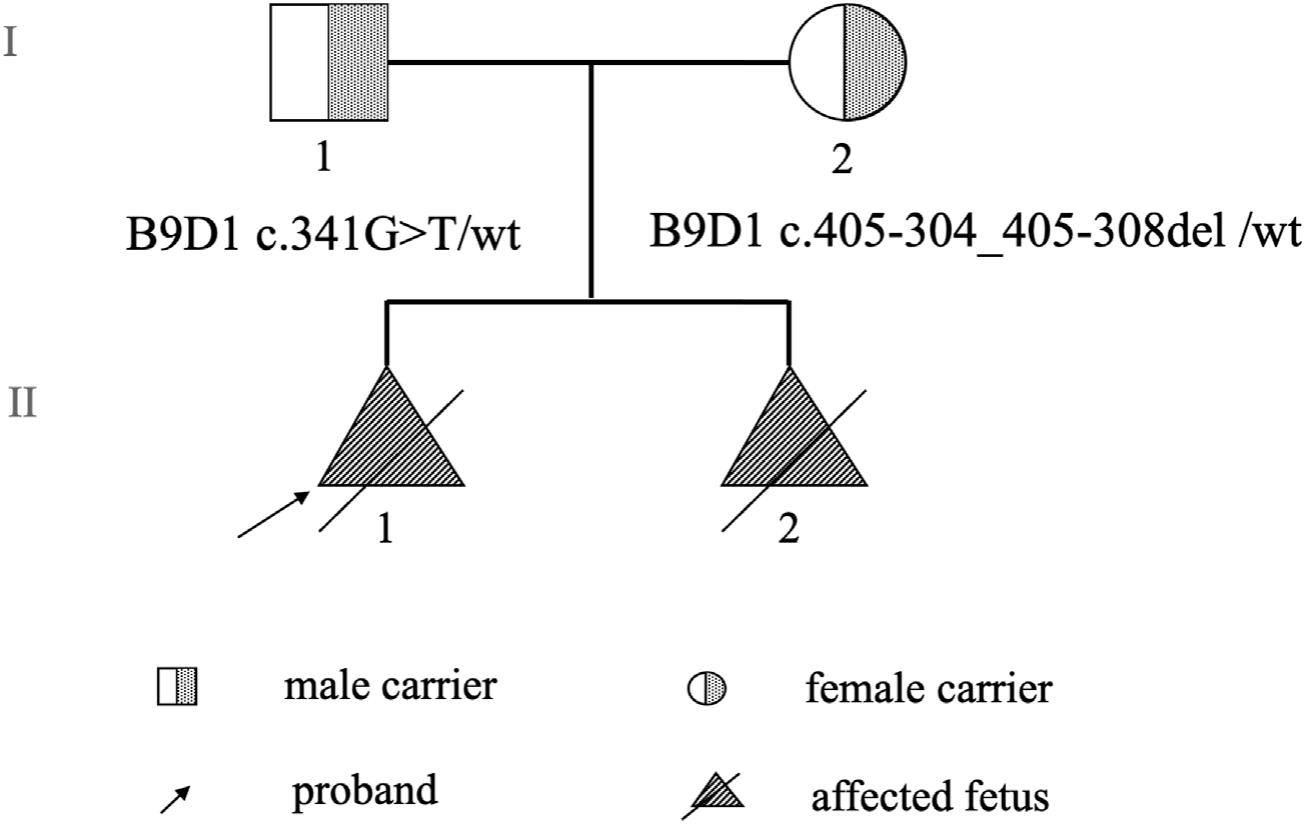
Pedigree chart.

Two cases of fetal malformation were noticed in the family. Both fetuses exhibited changes in fetal brain ultrasound at 24 weeks of pregnancy with phenotypes such as suspected Dandy–Walker malformation, meningoencephalocele, and polydactyly (toe) deformity. The mother was 37-year-old with a gravidity of 2 and parity of 0 (G2P0+2). In the first pregnancy, at 12 weeks, color Doppler ultrasound indicated “suspected fetal craniocele.” In the second trimester of pregnancy, amniotic fluid cells were subjected to high-throughput chromosomal sequencing and karyotype analysis, and no abnormalities were detected. At 24+ weeks of pregnancy, color Doppler ultrasound revealed “fetal head ultrasound changes, suspected Dandy–Walker malformation, hydrocephalus, meningoencephalocele, and suspected polydactyly (toe) deformity of both hands and feet.” Subsequently, the mother chose to terminate the pregnancy. The fetus was female, and no pathological anatomy was performed. In the second pregnancy, at 24+ weeks, color Doppler ultrasound revealed “fetal head ultrasound changes, suspected Dandy–Walker malformation, bilateral ventricular widening, meningocele, and fetal hands and feet ultrasound changes, suggesting polydactyly (toe) deformity.” Targeted magnetic resonance imaging of the fetal brain demonstrated “1. Bilateral cerebellar hemisphere separation, cerebellar vermis dysplasia, thin parenchyma, unclear display of the inferior vermis, expansion of the fourth ventricle, suggestive of a Dandy–Walker malformation; 2. Bilateral lateral ventricle dilatation, unclear display of the corpus callosum, and partial parallelism of the bilateral lateral ventricles, indicating the absence or dysplasia of the corpus callosum; 3. Partial defect of the occipital bone and meningocele beside it.” Pregnancy was terminated. The fetus was male with no pathological anatomy performed. No history of special medication, infection, or exposure to toxic substances was reported in both pregnancies, and no other complications was noticed during pregnancy. No family history of consanguineous marriage was reported (Figure 1).

### Results of WES and validation by Sanger sequencing

**FIGURE 2:**
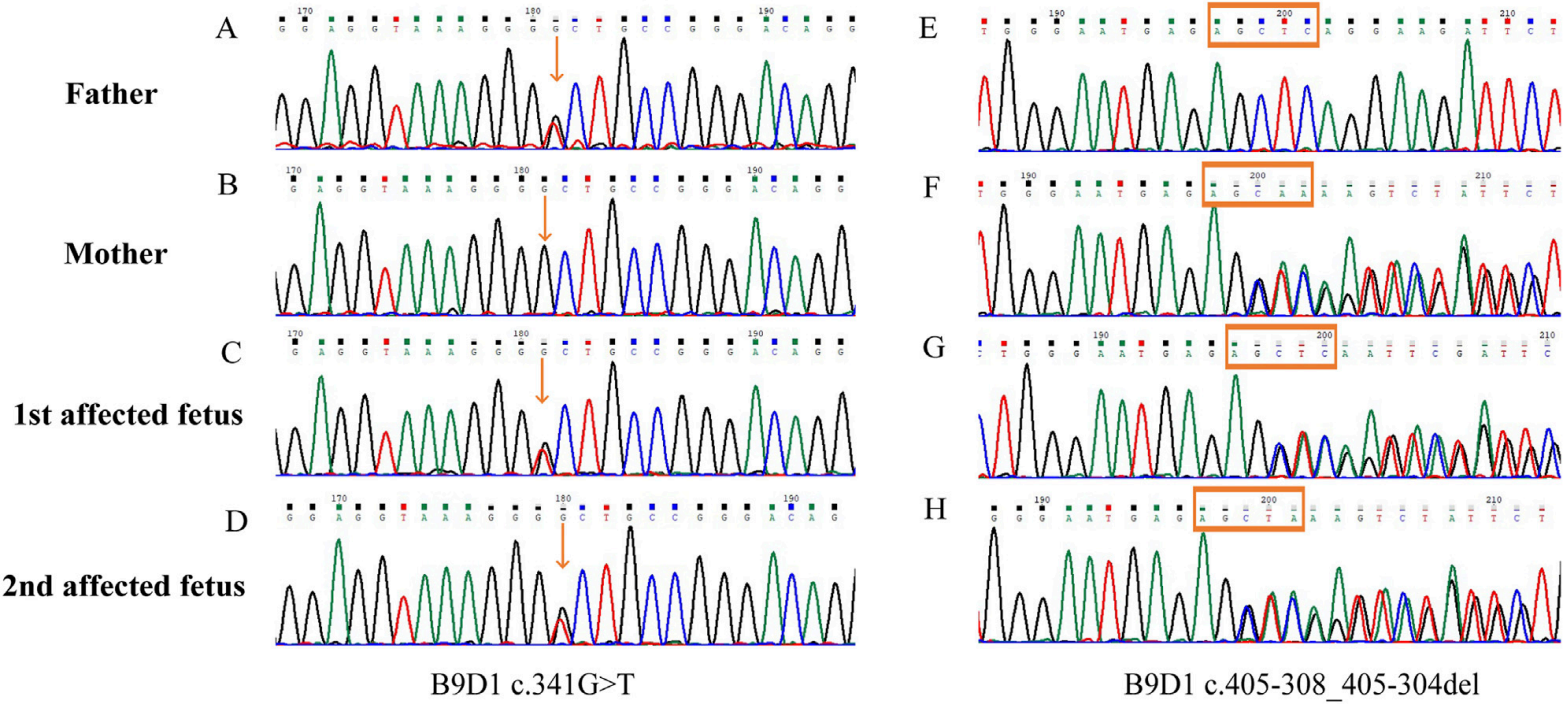
Sanger sequences of the B9D1 variants. **(A,E)** The missense variant (c.341G>T) and wild type c.405-308_405-304 detected in the father; **(B,F)** The wild type c.341 and deep-intronic variant c.405-308_405-304del detected in the mother; **(C,G)** Compound heterozygous B9D1 variants (c.341G>T and c.405-308_405-304del) in the first affected fetus; **(D,H)** Compound heterozygous B9D1 variants (c.341G>T and c.405-308_405-304del) in the second affected fetus.

The two affected fetuses were analyzed using WES in April and June 2021, respectively. The results did not reveal any specific pathogenic or likely pathogenic mutations. In July 2023, the WES datasets were reanalyzed. During reanalysis, the filtering parameters were reset to include suspicious disease-associated variants that had been previously filtered out as unqualified data because they were sequenced only a few times. Based on the clinical phenotype and genetic models of diseases, two previously filtered out variants of *B9D1* were discovered in both fetuses. Compound heterozygous variants (c.341G>T and c.405-308_405-304del, NM_015681.6) were suspected to be disease-associated variants in the probands. Parental Sanger sequencing showed that c.341G>T (p.R114L) was a paternal variant, and c.405-308_405-304del was a maternal variant (Figure 2).

### Bioinformatic analysis

**FIGURE 3:**
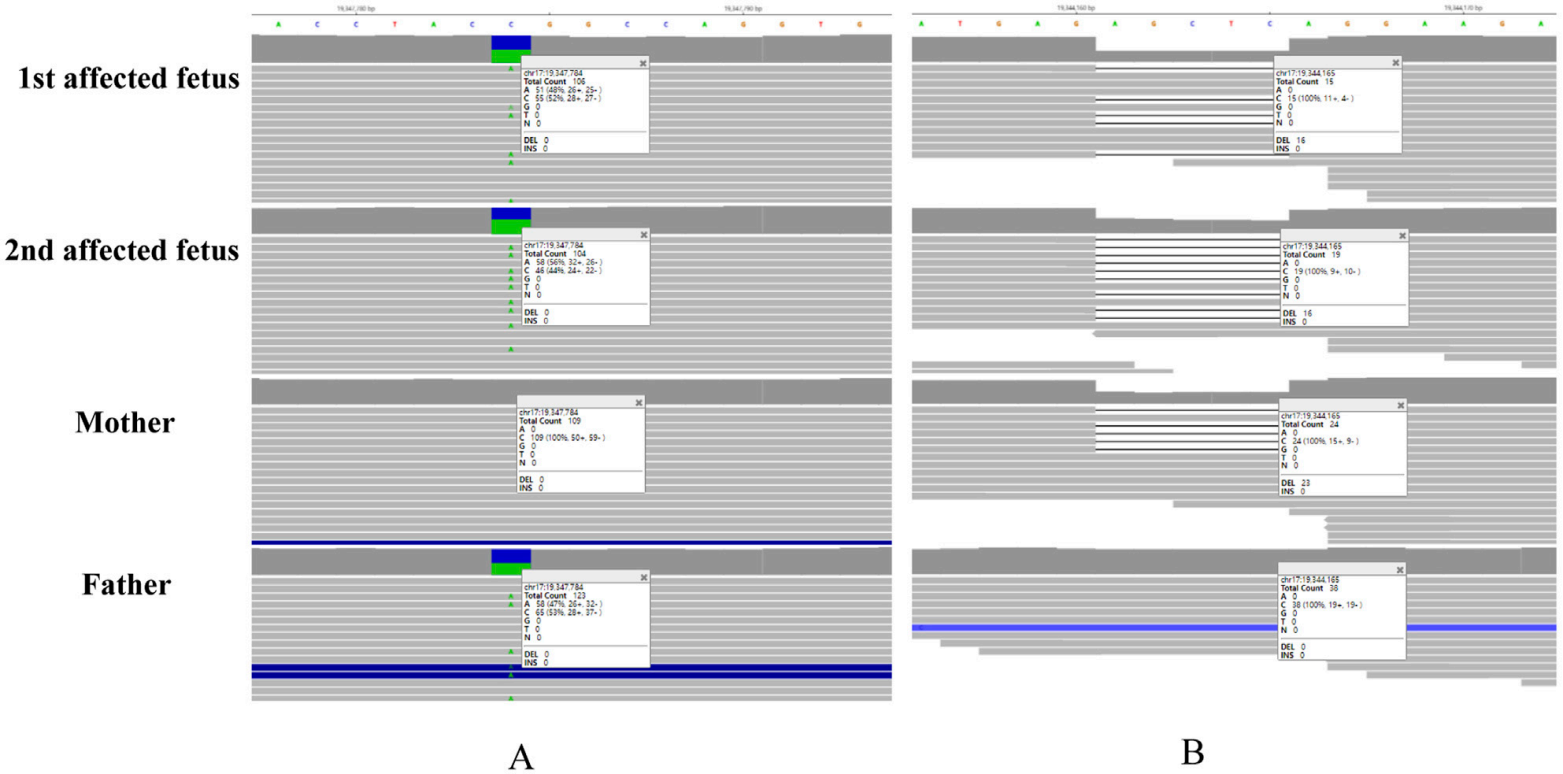
Integrative Genomics Viewer (IGV) screenshots of the B9D1 variants. **(A)** Paternal missense variant c.341G>T presented in the IGV, the sequencing depth of all samples reaches 100×. **(B)** Maternal deep-intronic variant c.405-308_405-304del presented in the IGV, the sequencing depth were significantly lower, range from 15× to 38×. The sequencing times for c.405-308_405-304 are significantly lower than the flanking base pairs.

The paternal variant c.341G>T (p.R114L) of *B9D1* was located in the last base pair of exon 4 in *B9D1*. It was predicted to be deleterious by CADD (CADD_Phred score, 34). Given that this variant was situated near the exon–intron junction, RNA splicing prediction tools were used to assess its potential impact on splicing. dbscSNV_ADA_SCORE (0.999) and dbscSNV_RF_SCORE (0.978) indicated a high probability of the variant being splice-altering. Meanwhile, the delta score in SpliceAI for the c.341G>T (p.R114L) variant was 0.57, suggesting that the tool predicted altered splicing of this variant. Based on the clinical phenotypic match, we strongly suspected that the autosomal recessive gene, *B9D1*, was causative. Maternal variants could not be identified because of technical limitations. Therefore, we screened the original sequencing data. Eventually, we discovered 5-bp base pairs (chr17:19344161-19344165), located in the intron between exons 5 and 6. The sequencing times for these base pairs were significantly shorter than those for the flanking base pairs (Figure 3). This condition was found in the maternal and affected fetal data but not in the paternal data. Therefore, the maternal variant c.405-308_405-304del, which was screened using a conventional bioinformatic pipeline, was identified. This maternal variant was located in an intron and was likely to affect alternative splicing. The SpliceAI delta score of the c.405-308_405-304del variant was 0.33 in the acceptor gain type.

### Splicing study of B9D1 c.341G>T by RT–PCR and Sanger sequencing

**FIGURE 4:**
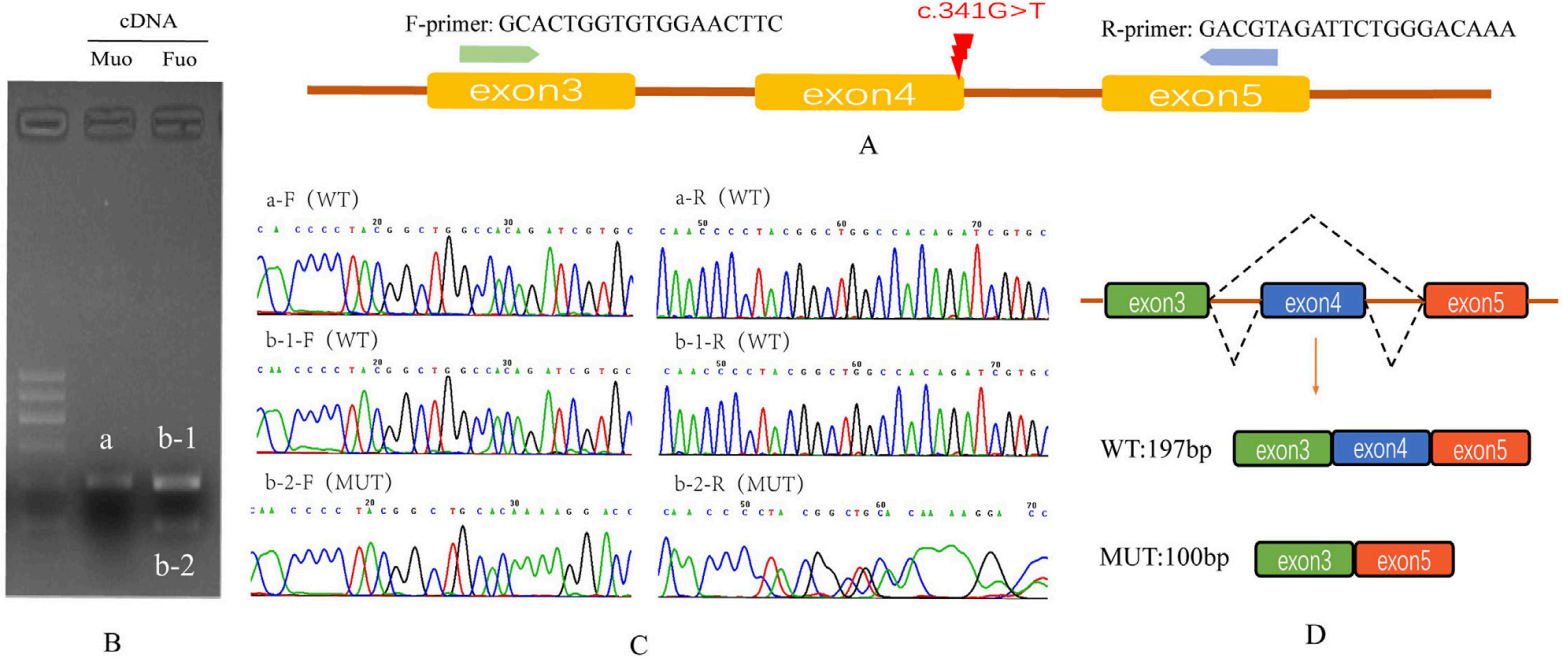
Splicing study of B9D1 c.341G>T by RT-PCR and Sanger’s Sequencing. **(A)** Schematic diagram of variant c.341G>T and designed flanking primers in the exon 3 and exon 5. **(B)** Agarose gel electrophoresis results of cDNA fragments from RT-PCR: the expected wild type fragment (a and b-1) is 197 bp, and the father had an extra mutation type fragment (b-2). **(C)** The Sanger sequencing of gel extraction product for the band a, b-1 and b-2. **(D)** Schematic of splicing for B9D1 c.341G>T, mutation type has a deletion of exon 4 (97 bp) in the B9D1 cDNA. Mu0, mother; Fu0, father; WT, wild type; MUT, mutation type.

The splicing consequence caused by B9D1 c.341G>T was verified through RT–PCR and Sanger sequencing of RNA derived from peripheral blood mononuclear cells of maternal and paternal blood (Figure 4). Agarose gel electrophoresis of the maternal RT–PCR products revealed a single band (Figure 4B, b,a). In contrast, the paternal products showed two bands (Figure 4B bands b-1 and b-2). The Sanger sequences revealed the wild-type (expected 197 bp) and mutant (expected 100 bp) splicing isoforms. The mutant isoform contained a deletion of exon 4 (97 bp) in *B9D1* cDNA from the c.341G>T variant. This was predicted to lead to an open reading frame change that affected 59.8% of the subsequent coding sequence.

### Splicing study of B9D1 c.405-308_405-304del by minigene assay

For the maternal variant, we initially employed the validation method same as that for the paternal variant. However, consistent or stable results were not obtained. Therefore, we used a minigene splicing assay for experimental validation.

**FIGURE 5:**
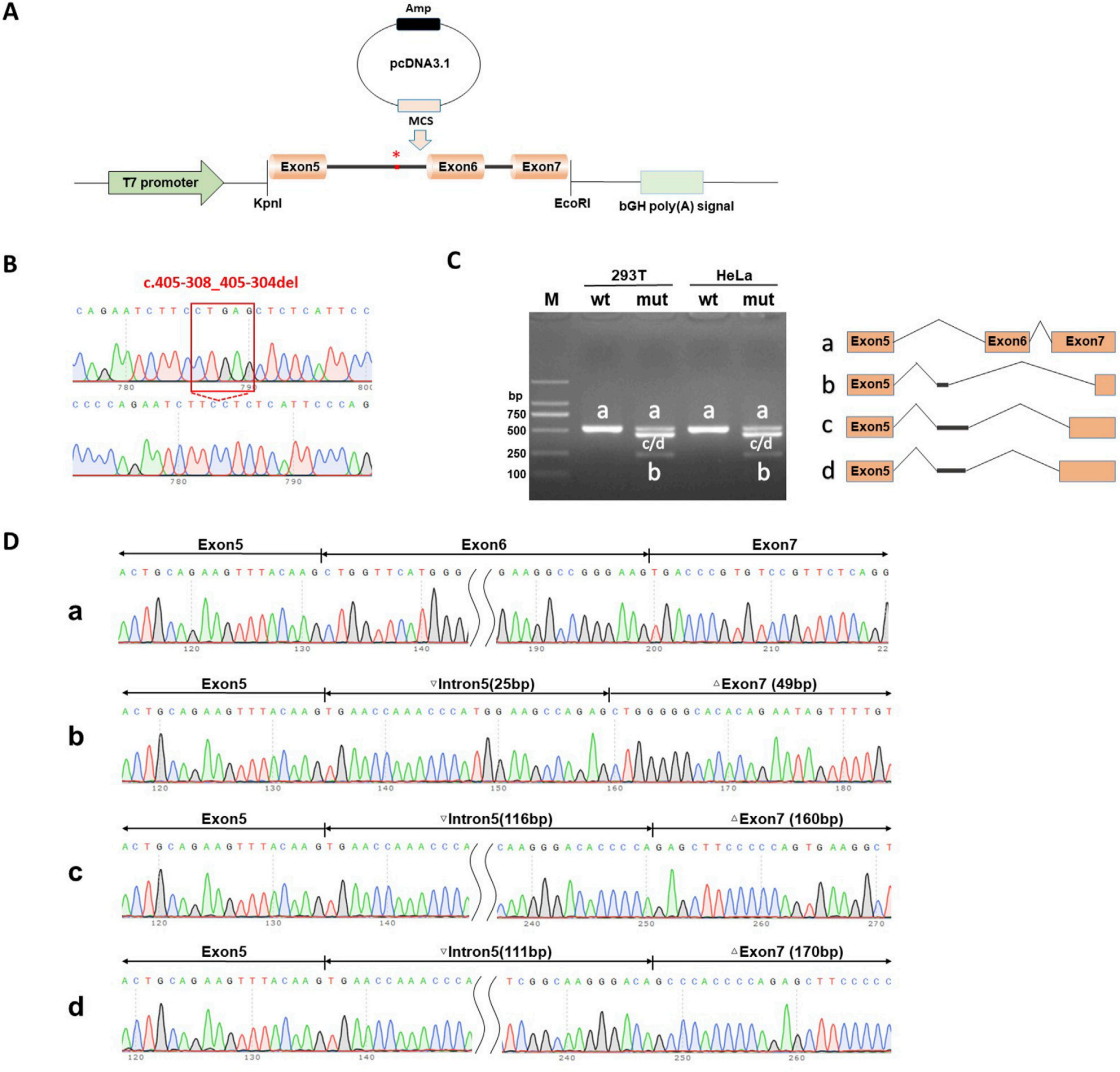
Splicing Study of B9D1 c.405-308_405-304del by minigene assay of the pcDNA3.1 vector. **(A)** Schematic diagram of pcDNA3.1 vector plasmids used in minigene assay. **(B)** Sanger sequencing validation of variant B9D1 c.405-308_405-304del and wild type recombinant plasmids. **(C)** Agarose gel electrophoresis results of RT-PCR in HeLa and 293T cells transfected wild-type recombinant plasmids was a single band (b and a), which was basically consistent with the expected size (527 bp). Meanwhile, the mutant type had four bands (bands a, b, c and d). **(D)** The Sanger sequencing of gel extraction product for the bands a, b, c and d. Sanger sequencing revealed that band-a were normal splicing bands, with a splicing pattern of Exon 5 (63 bp)-Exon 6 (68 bp)-Exon 7 (287 bp). The mutant band-b shows partial intron 5 (25 bp) retention and Exon 6 jump, along with a 238 bp deletion of Exon 7, with a splicing pattern of Exon 5 (63 bp)-▽Intron5 (25 bp)-△Exon 7 (49 bp). Mutant b and-c shows 116 bp intron5 retention and Exon 6 jump, along with a 127 bp deletion of Exon 7, with a splicing pattern of Exon 5 (63 bp)-▽Intron5 (116 bp)-△Exon 7 (160 bp). The splicing pattern of mutant b and-d is Exon 5 (63 bp)-▽Intron5 (111 bp)-△Exon 7 (170 bp).

The splicing results are shown in Figure 5. Wild-type splicing in HeLa and 293T cells produced a single band, consistent with the expected size (527 bp), and the mutant produced three bands. Sanger sequencing showed that the wild-type band was normally spliced with a splicing pattern of exon5 (63 bp)-exon6 (68 bp)-exon7 (287 bp). The mutant bands were abnormally spliced and exhibited three abnormal splicing patterns. Mutant band-b showed partial intron5 (25 bp) retention and exon6 jump, along with a 238-bp deletion of exon7, with a splicing pattern of exon5 (63 bp)-▽intron5 (25 bp)-△exon7 (49 bp). Mutant band-c showed 116-bp intron5 retention and exon6 jump, along with a 127-bp deletion of exon7, with a splicing pattern of exon5 (63 bp)-▽intron5 (116 bp)-△exon7 (160 bp). The splicing pattern of mutant band-d was exon5 (63 bp)-▽intron5 (111 bp)-△exon7 (170 bp).

The minigene assay demonstrated that the c.405-308_405-304del mutation affected the normal splicing of mRNA and generated several abnormal splicing isoforms. This *in vitro* assay revealed that the c.405-308_405-304del mutation resulted in a new and strong acceptor site at c.405-294_405-293. At the cDNA and protein levels, the expression patterns of the abnormal splicing isoforms led to a change in the subsequent reading frame and generated a stop codon, PTC, in advance, which might induce nonsense-mediated mRNA degradation (NMD) and produce truncated proteins of 159-amino acid/143-amino acid length.

### Analyzing variant pathogenicity according to American college of medical genetics (ACMG) guidelines

We demonstrated that the c. 341G > T (p. R114L) variant could lead to exon skipping (PVS1). The frequency of the c.341G>T (p.R114L) variant in the population database is zero (PM2_Supporting). Familial co-segregation occurs with diseases and mutations (PP1). According to the ACMG guidelines (Richards et al., 2015; Walker et al., 2023), the c.341G>T (p.R114L) variant (PVS1+PM2_Supporting+PP1) is classified as a pathogenic variant. Moreover, the c.405-308_405-304del variant has been shown to generate a stop codon, PTC, in advance, resulting in truncated proteins (PVS1). The frequency of this novel variant in the population database is zero (PM2_Supporting), and the variant from the father conforms to the genetic laws of recessive genetic diseases (PM3). According to the ACMG guidelines, the c.405-308_405-304del variant (PVS1+PM2_Supporting+PM3+PP1) is also classified as a pathogenic variant.

## Discussion

In this study, we report the case of a family with a recurrent history of a similar fetal anomaly. Both fetuses were diagnosed with Dandy–Walker malformation, meningoencephalocele, and polydactyly by prenatal ultrasound in the second trimester. Considering the family history and different fetal sexes, autosomal recessive Mendelian disorders were highly likely. However, two trio WES analyses were performed for the two affected fetuses without obtaining any meaningful findings. WES reanalysis can increase the diagnostic yield by 10%–20% in previously exome-negative individuals (Ewans et al., 2018; van Slobbe et al., 2024). Therefore, WES reanalysis and functional verification experiments were performed in this case, which revealed pathological compound heterozygous variants, paternal B9D1 c.341G>T and maternal B9D1 c.405-308_405-304del, in the two affected fetuses.

**FIGURE 6:**
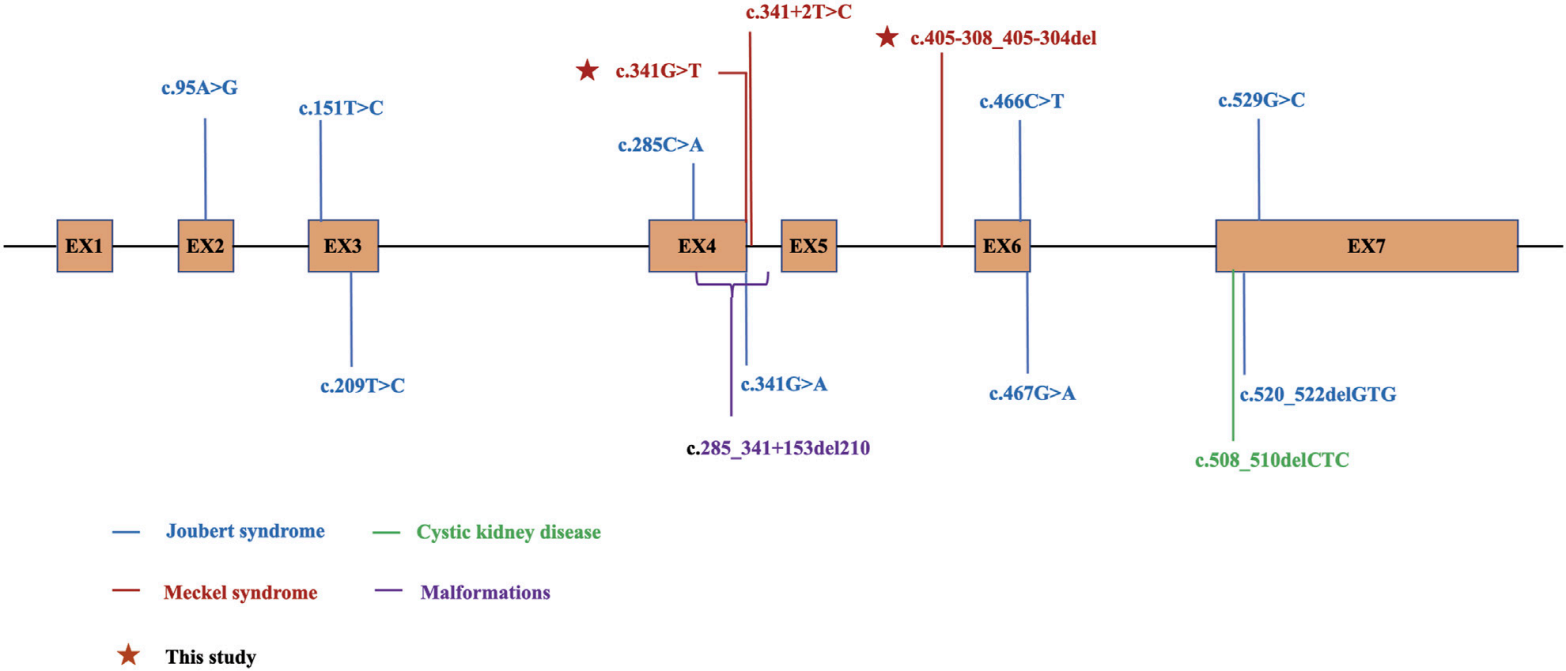
Schematic diagram for B9D1 and locations of DM mutations in HGMD. The variants with phenotype Joubert syndrome are shown in blue, Meckel syndrome in red, cystic kidney disease in green, malformations in purple, and the variants identified in this study are marked with stars. EX, exon.

B9D1 plays a pivotal role in cilia formation and retention, and may be involved in embryogenesis and developmental processes. However, the precise underlying mechanisms remain unclear. As of September 2024, 16 mutations in *B9D1* have been reported in the HGMD database, most of which are related to ciliary diseases and may lead to MKS. Ten of these mutations are related to ciliopathies including Meckel syndrome (MKS; also referred to as Meckel–Gruber syndrome, MIM249000) and Joubert syndrome (JS, MIM213300) (Figure 6). *B9D1* was identified as a novel MKS gene in 2011 (Hopp et al., 2011). MKS is a rare autosomal recessive disorder characterized by a combination of severe congenital anomalies, including abnormalities in the central nervous system (typically occipital meningoencephalocele and posterior fossa anomaly), kidneys (typically cystic dysplasia), liver (such as ductal plate malformations), and postaxial polydactyly. However, the high variability of clinical manifestations in MKS and specific combination and severity of these anomalies that vary among individuals can make diagnosis difficult (Maglic et al., 2016; Stembalska et al., 2021).

Notably, neither affected fetus in our study showed detectable renal abnormalities on prenatal ultrasound, despite renal involvement being one of the most typical features of MKS. This observation raises important questions regarding phenotypic variability and differential diagnosis, particularly with JS and other ciliopathies that may present with overlapping CNS malformations but milder or absent renal involvement. The severity of CNS defects (e.g., occipital encephalocele, Dandy-Walker malformation) support the diagnosis of our cases as MKS rather than JS. Meanwhile, this apparent unusual feature may reflect several factors involved. First, prenatal imaging has recognized limitations in detecting subtle renal pathology, particularly the microscopic cystic changes of MKS that often develop progressively during gestation. Second, the timing of imaging (24 weeks in our cases) may have preceded the full manifestation of renal abnormalities. Third, our case might be a distinct clinical entity at the MKS-JS spectrum borderline. The absence of postmortem examinations represented an unavoidable limitation of our phenotypic characterization. This underscores the importance of postmortem evaluations in future cases to better understand potential genotype-phenotype correlations and the full spectrum of organ involvement in B9D1-related ciliopathies.

We identified a paternal variant of *B9D1* in both fetuses. This variant was a missense mutation located at the last base of exon 4 in *B9D1* Katiyar et al. have reported a different missense mutation variant at the same base that translated into a different amino acid (c.341G>A, p. R114Q). This variant abolished the 5′-splice site of intron 4 of *B9D1*, resulting in out-of-frame skipping of exon 4 (r.245_341del; p. Trp82Cysfs*45) (Katiyar et al., 2020). Hopp et al. have reported a single fetus affected by MKS, who was heterozygous for the typical splicing mutation c.505+2T>C in *B9D1*, resulting in the skipping of exon 4 (Hopp et al., 2011). In our study, RT-PCR demonstrated the skipping of exon 4 caused by c. 341G > T (p. R114L), which was consistent with the splicing results above. This further confirmed the judgment of pathogenicity.

After confirming the pathogenicity of the paternally derived variant in *B9D1*, we attempted to identify potential maternally derived variants. The maternal variant *B9D1* c.405-308_405-304del was previously filtered out as unqualified in the analysis pipeline owing to the lack of sequencing depth. By adjusting the FisherStrand filter threshold in the Genome Analysis Toolkit (GATK) from >100 to >120, we identified a maternally derived variant c.405-308_405-304del. This finding was subsequently validated using the Integrative Genomics Viewer (IGV) and Sanger sequencing results. This variant was a deep-intronic variant, located in the intron region between exons 5 and 6, far from the exon boundary. Deep-intronic variants are generally considered to not directly affect the coding sequences of proteins (Vaz-Drago et al., 2017). Instead, they may affect RNA splicing by creating new splice sites, enhancing or inhibiting the existing splice sites, and altering the binding of splice regulators (Kurosawa et al., 2023). These splicing alterations may lead to mRNA destabilization by NMD or functional defects in the encoded proteins, thereby indirectly affecting protein structure and function. While our data suggest that the deep-intronic variant c.405-308_405-304del generates aberrant transcripts harboring a PTC in advance, which is typically targeted by NMD, we acknowledge that this is based solely on prediction without direct experimental validation. Due to limitations in sample availability, we did not perform functional assays to confirm NMD activity. Future studies using patient-derived cellular models or minigene splicing assays coupled with NMD inhibition would be valuable to experimentally validate this mechanism. Evaluating the pathogenicity of deep intronic variants is challenging owing to the specificity of their location and complexity of their underlying mechanisms. We validated the effect of the deep-intronic variant c.405-308_405-304del of *B9D1* on splicing. The deep-intronic variant resulted in a new and strong acceptor site at c.405-294_405-293.

This study has some limitations. First, our study was conducted under specific laboratory conditions, which may not fully mimic the complex conditions in the actual clinical scenario. Second, while our study demonstrated that both B9D1 variants lead to aberrant splicing, we lack the direct evidence of protein-level for truncated isoforms. Due to limitations in sample availability, we were unable to perform Western blotting or other proteomic analyses to confirm whether these aberrant transcripts were translated into truncated proteins or underwent rapid degradation. Further experimental validation at the protein level including animal experiment is warranted.

## Conclusion

We discovered a compound heterozygous pathogenesis of *B9D1* in a recurrent MKS pedigree. This study revealed a novel pathogenic deep-intronic variant of *B9D1* and enhanced our understanding of the complex genetic basis of MKS. Further functional experiments using animal models are necessary for exploring the pathogenic mechanism in depth. Enhancing our understanding and diagnostic ability for this disease is essential for improving reproductive outcomes and reducing birth defects for affected individuals and their families.

## Data Availability

The variation data reported in this paper have been deposited in the Genome Variation Map (GVM) in National Genomies Data Center, Beijing Institute of Genomics. Chinese Academy of Sciences and China National Center for Bioinformation, under accession number GVM001124 (https://ngdc.cncb.ac.cn/gvm/).
